# A Novel Method of Serum Resistance by Escherichia coli That Causes Urosepsis

**DOI:** 10.1128/mBio.00920-18

**Published:** 2018-06-26

**Authors:** Carrie F. Coggon, Andrew Jiang, Kelvin G. K. Goh, Ian R. Henderson, Mark A. Schembri, Timothy J. Wells

**Affiliations:** a The University of Queensland Diamantina Institute, The University of Queensland,Translational Research Institute, Brisbane, Queensland, Australia; b School of Chemistry and Molecular Biosciences, The University of Queensland, Brisbane, Queensland, Australia; c Australian Infectious Diseases Research Centre, University of Queensland, Brisbane, Queensland, Australia; d Institute of Microbiology and Infection, The University of Birmingham, Birmingham, United Kingdom; Washington University School of Medicine

**Keywords:** *Escherichia coli*, antibody function, lipopolysaccharide, sepsis, serum resistance

## Abstract

Uropathogenic Escherichia coli (UPEC) is the most common cause of urinary tract infection, which in some patients can develop into life-threatening urosepsis. Serum resistance is a key virulence trait of strains that cause urosepsis. Recently, we identified a novel method of serum resistance in patients with Pseudomonas aeruginosa lung infections, where patients possessed antibodies that inhibited complement-mediated killing (instead of protecting against infection). These inhibitory antibodies were of the IgG2 subtype, specific to the O-antigen component of lipopolysaccharide (LPS) and coated the bacterial surface, preventing bacterial lysis by complement. As this mechanism could apply to any Gram-negative bacterial infection, we hypothesized that inhibitory antibodies may represent an uncharacterized mechanism of serum resistance in UPEC. To test this, 45 urosepsis patients with paired blood culture UPEC isolates were screened for serum titers of IgG2 specific for their cognate strain’s LPS. Eleven patients had sufficiently high titers of the antibody to inhibit serum-mediated killing of UPEC isolates by pooled healthy control sera. Depletion of IgG or removal of O-antigen restored sensitivity of the isolates to the cognate patient serum. Importantly, the isolates from these 11 patients were more sensitive to killing by serum than isolates from patients with no inhibitory antibodies. This suggests the presence of inhibitory antibodies may have allowed these strains to infect the bloodstream. The high prevalence of patients with inhibitory antibodies (24%) suggests that this phenomenon is an important mechanism of UPEC serum resistance. LPS-specific inhibitory antibodies have now been identified against three Gram-negative pathogens that cause disparate diseases.

## IMPORTANCE

Despite improvements in the early detection and management of sepsis, morbidity and mortality are still high. Infections of the urinary tract are one of the most frequent sources of sepsis with Escherichia coli the main causative agent. Serum resistance is vital for bacteria to infect the bloodstream. Here we report a novel method of serum resistance found in patients with UPEC-mediated sepsis. Antibodies in sera usually protect against infection, but here we found that 24% of patients expressed “inhibitory antibodies” capable of preventing serum-mediated killing of their infecting isolate. Our data suggest that these antibodies would allow otherwise serum-sensitive UPEC strains to cause sepsis. The high prevalence of patients with inhibitory antibodies in this cohort suggests that this is a widespread mechanism of resistance to complement-mediated killing in urosepsis patients, invoking the potential for the application of new methods to prevent and treat sepsis.

## OBSERVATION

Urinary tract infections (UTIs) are one of the most common human infections. They can affect the bladder (cystitis), kidneys (pyelonephritis), and can lead to bloodstream infection (urosepsis). UTIs account for roughly 9% of severe sepsis cases (1), with uropathogenic Escherichia coli (UPEC) as the most common cause (2). UPEC isolated from patients with pyelonephritis exhibit much higher serum resistance (82 to 93%) than fecal E. coli isolates (57%) (3), and mechanisms that allow these strains to resist the bactericidal activity of human serum are key virulence traits for the development of urosepsis (4, 5).

Recently, we described a novel mechanism of serum resistance for Pseudomonas aeruginosa where specific antibody, instead of targeting the bacteria for destruction, protected the bacteria from complement-mediated lysis (6). These “inhibitory antibodies” were present in patient serum at high titers, were of the IgG2 subtype, and specifically recognized the O-antigen component of lipopolysaccharide (LPS). Our results suggested that “inhibitory antibodies” prevented complement-mediated lysis by binding at high density to the O-antigen, a target distal from the cell surface, and sterically blocking access of complement to the cell membrane. Patients with inhibitory antibodies had worse lung function than patients with normal serum killing, and removal of these antibodies by plasmapheresis ameliorated infection-related symptomology (7).

In addition to the observations above, we described similar inhibitory antibodies in a subset of HIV-positive patients that had high titers of IgG specific for the LPS of Salmonella enterica serovar Typhimurium (8). On the basis of these observations, we hypothesized that this serum resistance mechanism could apply to any Gram-negative bacterial infection where specific antibody to O-antigen is likely to be induced. Indeed, a 56°C heat-stable serum factor in patients with UTIs that prevents bactericidal killing by complement has already been reported (9, 10). Therefore, we sought to determine whether inhibitory antibodies exist in patients presenting with urosepsis. Here, we analyzed a panel of patients with UPEC-mediated urosepsis and identified the presence of inhibitory antibodies in approximately one-quarter of these individuals. On the basis of these observations, we suggest that inhibitory antibodies represent a widespread mechanism for bacterial survival in the bloodstream.

We wished to determine whether patients with UPEC-mediated urosepsis had O-antigen-specific inhibitory antibodies that might contribute to bacterial survival in the bloodstream. Therefore, we obtained serum samples from 45 patients with urosepsis (aged 33 to 98 years; 61% female), and the corresponding UPEC isolate recovered from the bloodstream of each patient. To test whether these strains produced O-antigen, we prepared LPS from all isolates and analyzed the preparations by silver staining (11). This revealed that 38 strains possessed detectable long-chain O-antigen (see Fig. S1 in the supplemental material).

10.1128/mBio.00920-18.1 FIG S1  The majority of E. coli isolates have high O-antigen expression. Lipopolysaccharide was purified from E. coli strains isolated from blood samples from 45 urosepsis patients, run on a 4 to 12% bis-Tris gel, and silver stained. O-antigen expression was detected in 38 of 45 strains. Download FIG S1, TIF file, 2.5 MB. Copyright © 2018 Coggon et al. 2018 Coggon et al. This content is distributed under the terms of the Creative Commons Attribution 4.0 International license.

**FIG 1:**
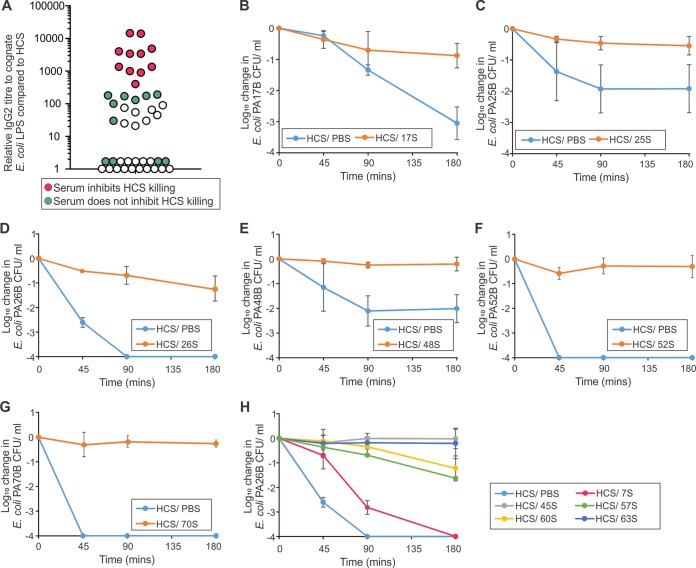
(A) Titers of IgG2 in patient sera specific for the LPS of their cognate UPEC strain relative to pooled healthy control sera (HCS). Sera that either inhibited (red circles) or did not inhibit (green circles) the bactericidal activity of pooled HCS when mixed 50:50 are indicated. Sera not tested for inhibition are indicated by white circles. (B to H) HCS mixed 50:50 with PBS (HCS/PBS) can kill UPEC strains PA17B (B), PA25B (C), PA26B (D), PA48B (E), PA52B (F), PA70B (G), and PA26B (H). Mixing this HCS 50:50 with patient serum 17S (HCS/17S) (B), 25S (C), 26S (D), 48S (E), 52S (F), 70S (G) and with 7S, 45S, 57S, 60S, and 63S (H) inhibits bacterial killing.

LPS-specific IgG2 is associated with inhibition of complement-dependent bacterial killing. However, the ability of IgG2 antibodies to inhibit killing is titer-dependent (7). Therefore, for each patient we measured the titer of IgG2 in the serum that was specific to the LPS isolated from their cognate infecting strain. Twenty-six of the 45 patient sera had an LPS-specific IgG2 titer higher than pooled healthy control sera (HCS) (Fig. 1A). Eleven sera had LPS-specific IgG2 titer of >180, potentially high enough to inhibit serum killing (7).

The presence of inhibitory antibodies can be confirmed by mixing patient serum with HCS and demonstrating in a serum bactericidal assay (SBA) that this mixture is poorer at bacterial killing than the HCS alone (6). Sera from 22 patients were tested in this manner: 17 with IgG2 titers of >100 and 5 with titers of <30 (Fig. 1A). Fourteen of the 22 matched isolates were resistant to HCS killing, so these sera were tested on HCS-sensitive strains with either the same or cross-reacting O-antigen serotype. Eleven sera with a titer of >400 could inhibit the bactericidal activity of HCS, either against the patient’s cognate isolate (Fig. 1B to G) or an appropriate serum-sensitive E. coli strain (Fig. 1H). In contrast, sera with a titer of <190 could not inhibit serum-mediated killing (Fig. S2). Thus, 11 of 45 urosepsis patients had serum that could significantly block the bactericidal activity of HCS (Fig. S2A), correlating with titers of O-antigen-specific antibody of >400. No serotype- or sequence type-specific associations were noted; however, sequence type 73 was overrepresented in patients with inhibitory antibodies (*P* < 0.05) (Table S1). All strains belonged to E. coli phylogroup B2 or D.

10.1128/mBio.00920-18.2 FIG S2  (A) Area under the curve results for all mixing SBAs performed. The area under the curve was calculated for HCS mixed 50:50 with PBS (green circles) and HCS mixed 50:50 with patient sera (red circles). Bars represent standard errors of three experiments. Patient serum was considered inhibitory if the area under the curve (AUC) was significantly higher than HCS/PBS killing. (B) HCS mixed 50:50 with PBS can kill UPEC strains PA6B (B), PA8B (C), PA26B (D), PA52B (E), and PA70B (F). Mixing this HCS 50:50 with patient serum 6S (B), 8S (C), 19S, 32S, 41S, 50S, and 55S (D), 67S (E), and 10S, 11S, and 28S (F) does not inhibit bacterial killing. Download FIG S2, TIF file, 13.8 MB. Copyright © 2018 Coggon et al. 2018 Coggon et al. This content is distributed under the terms of the Creative Commons Attribution 4.0 International license.

10.1128/mBio.00920-18.4 TABLE S1  Strain and titer information. Download TABLE S1, DOCX file, 0.02 MB. Copyright © 2018 Coggon et al. 2018 Coggon et al. This content is distributed under the terms of the Creative Commons Attribution 4.0 International license.

**FIG 2:**
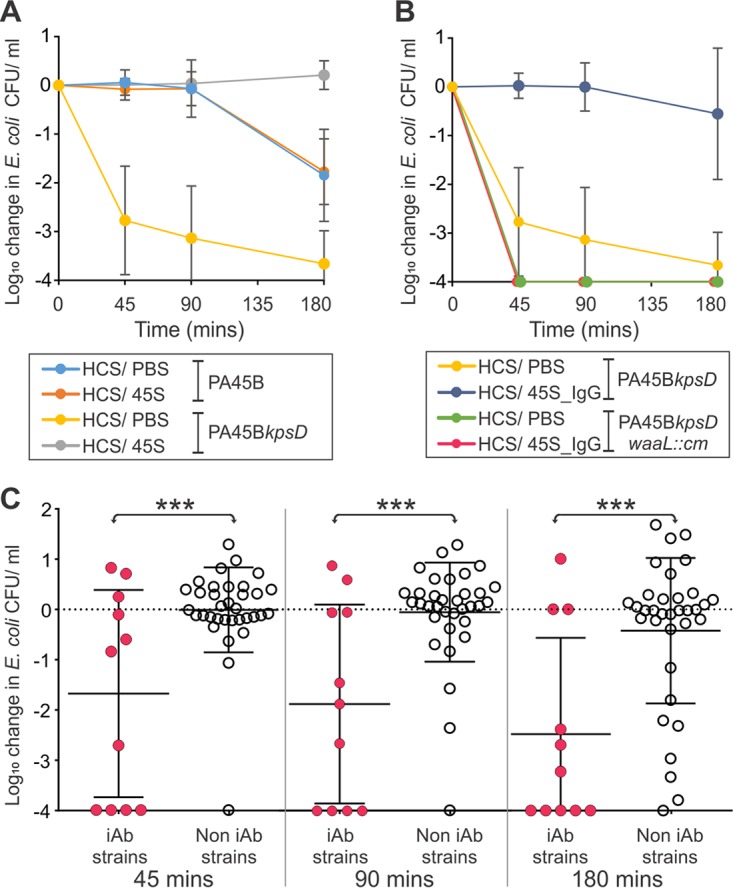
(A) HCS mixed 50:50 with PBS or 45S serum could not kill wild-type PA45B. In contrast, HCS mixed 50:50 with PBS led to killing of the PA45B*kpsD* capsule mutant. Addition of 45S to HCS inhibited the killing of PA45B*kpsD* strain. (B) Purified IgG from 45S when added to HCS 50:50 leads to complete inhibition of HCS-PBS killing. In contrast, both HCS/PBS and HCS/purified IgG mixes lead to complete killing of the O-antigen, capsule double mutant PA45B*kpsD waaL*::*cm*. (C) The bactericidal killing of all 45 UPEC isolates in undiluted HCS after either a 45-, 90-, or 180-min incubation. Strains isolated from patients possessing inhibitory antibodies in their serum (red circles) were significantly more sensitive to HCS than UPEC strains isolated from patients without inhibitory antibodies (white circles). iAb, inhibitory antibody. ***, *P* < 0.001.

Many of the E. coli isolates produced capsule, including some from patients with inhibitory antibodies (PA26B, PA45B, and PA63B). To determine whether capsule had any role in the antibody-mediated protection from serum killing, we performed an SBA on strain PA45B and its isogenic capsule mutant PA45B*kpsD* (Fig. 2A). In contrast to the wild type, the capsule mutant strain PA45B*kpsD* was killed in diluted HCS. However, when the autologous patient serum was mixed 50:50 with HCS, killing of PA45B*kpsD* was completely inhibited (Fig. 2A). Thus, capsule is not required for the protection mediated by PA45 serum (45S). To confirm that antibodies were responsible for the inhibition of HCS killing, we purified IgG from 45S. The addition of purified 45S IgG alone inhibited the killing of PA45B*kpsD* strain by HCS (Fig. 2B). In contrast, 45S depleted of antibody did not inhibit HCS killing (Fig. S3). To confirm that O-antigen was required for the antibody-mediated inhibition, we created a capsule, O-antigen double mutant, PA45B*kpsD waaL*::*cm*. In contrast to the O-antigen-positive strain, addition of either 45S or purified IgG from 45S was unable to inhibit HCS killing (Fig. 2B). Thus, IgG specific for the O-antigen is responsible for inhibition of serum killing.

10.1128/mBio.00920-18.3 FIG S3  PA45 serum depleted of IgG does not inhibit serum-mediated killing. HCS mixed 50:50 with PBS led to killing of both the capsule mutant PA45B*kpsD* and double mutant PA45B*kpsD waaL*::*cm*. Addition of 45S depleted of IgG antibody did not inhibit the killing of either mutant strain. Download FIG S3, TIF file, 2.1 MB. Copyright © 2018 Coggon et al. 2018 Coggon et al. This content is distributed under the terms of the Creative Commons Attribution 4.0 International license.

The assays above determined that some of the UPEC isolates tested (8/22) were sensitive to HCS killing, even though they were isolated from blood. We therefore examined all 45 isolates for sensitivity to killing by HCS. In these experiments, the 11 strains isolated from patients with inhibitory antibody were significantly more sensitive to killing by HCS over a 3-h incubation period than strains from patients that did not have high-titer inhibitory antibody (*P* < 0.001) (Fig. 2C). Thus, we conclude that the presence of inhibitory antibody is frequently associated with serum-sensitive UPEC bloodstream isolates.

The ability to resist the bactericidal effect of serum is a key virulence trait for the survival of UPEC in the bloodstream (3–5, 12). The O-antigen represents a major mechanism of UPEC serum resistance (13), but other factors, including capsule, also contribute to this phenotype (12–14). Thus, the production of O-antigen is not uniquely associated with protection from complement-mediated killing. Here we found that 24% of urosepsis patients had inhibitory antibodies, indicating that this may represent an important mechanism by which some UPEC strains exhibit resistance to complement-mediated killing. Indeed, the majority of strains isolated from patients with inhibitory antibodies (8/11) were sensitive to HCS, suggesting that these strains require the presence of inhibitory antibodies to survive in the bloodstream. One strain (PA45B) was serum-sensitive in HCS only after deletion of capsule; however, the presence of inhibitory antibodies in the patient serum afforded full protection against complement-mediated killing even in the mutant strain. Thus, these antibodies can protect bacteria from serum killing regardless of capsule production.

A blocking factor of complement-mediated killing in patients with UTIs has previously been reported. In this 1966 study, nine patients with chronic pyelonephritis possessed serum that blocked the ability of HCS to kill their cognate strain in a strain-specific manner (9). Similarly, in a study from 1972, 9 of 48 patients with UTIs possessed serum that could not kill their HCS-susceptible cognate strain; however, the serum could kill a known susceptible E. coli (10). Strains isolated from patients with this defect included E. coli, Proteus morganii, Proteus mirabilis, and *Klebsiella* spp. (10). The serum defect was studied in detail for the two *Proteus* species and found to be IgG specific for the LPS of each of the strains (15). In light of our new findings, we believe it is likely that the patients in these studies also possessed inhibitory IgG2 specific for the O-antigen of LPS. If this is correct, it strengthens the hypothesis that inhibitory antibodies are common in patients that suffer UTIs, and these antibodies may exist for multiple Gram-negative species. Indeed, having previously identified similar antibodies against P. aeruginosa and S. Typhimurium, this study demonstrates that inhibitory antibodies exist for an even wider range of Gram-negative bacteria.

In the studies described above, the serum-blocking factor disappeared following successful treatment and eradication of the infecting organism (9, 10). Therefore, the presence of the infecting organism seems necessary to maintain high titers of inhibitory antibody, and eradication of the bacteria by antibiotic treatment may lead to subsequent loss of deleterious antibody. Additionally, we had recent success in treating bronchiectasis patients who have inhibitory antibodies with plasmapheresis, where all antibodies were removed from the serum and replaced with donor intravenous immunoglobulin, leading to improvement in health and the loss of culturable P. aeruginosa in the sputum (7). This suggests that removing the inhibitory antibody in patients with urosepsis, via plasmapheresis or other methods, may be an option to combat infections caused by multidrug-resistant pathogens that do not respond to antibiotic treatment.

### Methods.

The paired plasma and UPEC blood culture isolates collected from the 45 urosepsis patients have been described previously (2). *In silico* serotyping was performed using the online tool SerotypeFinder 1.1 (16). The capsule mutant strain PA45B*kpsD* was described in a previous study (14). The capsule, O-antigen double mutant strain PA45B*kpsD waaL*::*cm* was constructed as previously described (14). Strains were grown in solid or liquid lysogeny broth (LB) at 37°C. This study was performed in accordance with the ethical standards of The University of Queensland, Princess Alexandra Hospital, and the Helsinki Declaration. The study was approved, and the need for informed consent was waived by the institutional review board of the Princess Alexandra Hospital (2008/264).

LPS was extracted from UPEC strains as previously described (11). Extracts were analyzed on a 4 to 12% Bolt gel (Life Sciences) with silver staining (Thermo Scientific). LPS was quantified by comparison to five standards (10, 5, 1, 0.5, and 0.1 mg/ml) of commercially available E. coli LPS (Sigma). LPS-specific IgG2 titer was measured as previously described with the following modifications (6). Patient sera were tested against the cognate strain LPS unless otherwise indicated. HCS was pooled from at least three healthy donors and used as a negative control in enzyme-linked immunosorbent assays (ELISAs). The wells on the plates were coated with 1 µg/ml of the appropriate LPS. Patient sera were diluted across the plate (1:20 to 1:14,580). The secondary antibody was monoclonal anti-human IgG2 (Fc specific) (diluted 1:2,000; Sigma-Aldrich) followed by a tertiary antibody of alkaline phosphatase-labeled anti-mouse IgG (diluted 1:10,000). Absorbance readings were measured after 15-min incubation with the substrate. Antibody titer was determined relative to pooled HCS. The sample cutoff was the average negative response plus 3 standard deviations. IgG was purified from serum using a protein G column as previously described (6). Purified IgG was concentrated to be equivalent to the initial serum.

Serum bactericidal assays (SBAs) were performed in triplicate as described previously (6). Serum or purified IgG was either undiluted or mixed 50:50 with either phosphate-buffered saline (PBS) or HCS. Serum heated to 56°C for 20 min had no bacterial killing activity and was used as a control. Statistical differences between samples were determined by comparing log_10_ change in killing and area under the curve via a Student’s *t* test. Analysis of the observed distribution of sequence types was performed using a Wilson/Brown binomial test.
