# Supplementary figures and images for: A Novel Method of Serum Resistance by Escherichia coli That Causes Urosepsis
Source: mBio. 2018 Jun 26;9(3):e00920-18. doi: 10.1128/mBio.00920-18 (PMC6020292; doi:10.1128/mBio.00920-18)

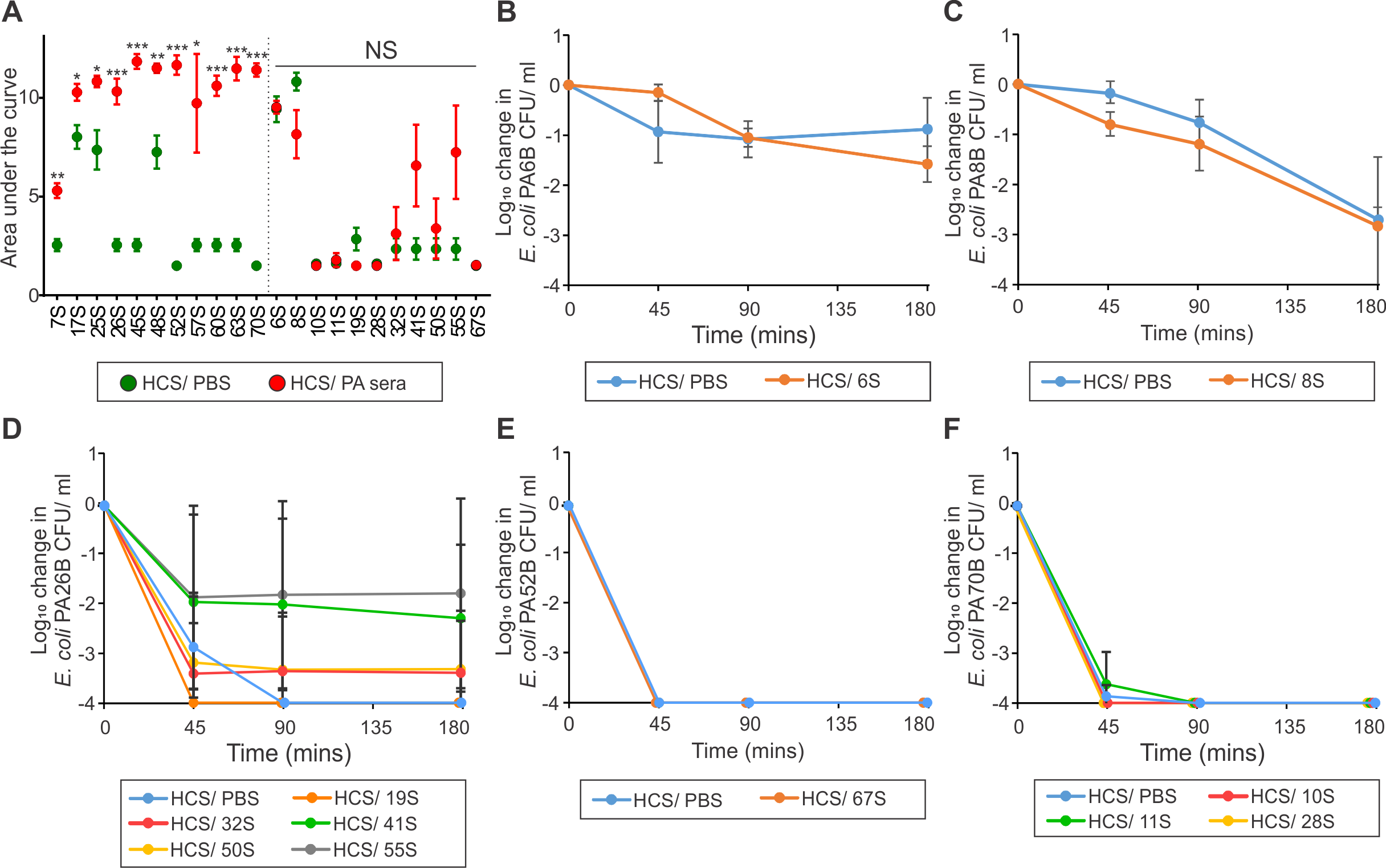

Supplement: FIG S2 [file mbo003183946sf2.tif]

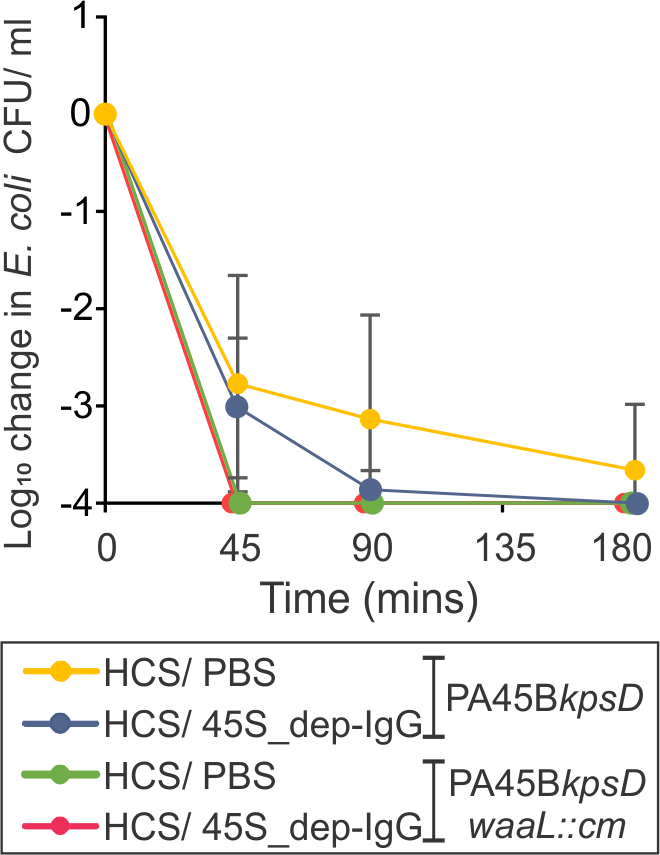

Supplement: FIG S3 [file mbo003183946sf3.tif]
