# Supplementary material for: A Novel Method of Serum Resistance by Escherichia coli That Causes Urosepsis
Source: mBio. 2018 Jun 26;9(3):e00920-18. doi: 10.1128/mBio.00920-18 (PMC6020292; doi:10.1128/mBio.00920-18)
Supplement: TABLE S1 [file mbo003183946st1.docx]

Table S1. Strain and titre information.

| **Strain** | **Sequence Type** | **O serotype** | **Phylogroup** | **Anti-LPS IgG2 titre** |
| --- | --- | --- | --- | --- |
| PA05B | 12 | 4 | B2 | >20 |
| PA06B | 405 | 102 | D | >20 |
| PA07B | 73 | 6 | B2 | 900 |
| PA08B | 95 | 1 | B2 | 190 |
| PA09B | 38 | 86 | D | >20 |
| PA10B | 95 | 1 | B2 | 170 |
| PA11B | 95 | 1 | B2 | 30 |
| PA15B | 12 | 4 | B2 | >20 |
| PA17B | 69 | 17/77 | D | 14580 |
| PA19B | 73 | 25 | B2 | >20 |
| PA20B | 69 | 17/77 | D | 55 |
| PA21B | - | 17/77 | D | >20 |
| PA22B | 12 | 4 | B2 | 75 |
| PA25B | 12 | 4 | B2 | 1350 |
| PA26B | 73 | 2 | B2 | 1350 |
| PA28B | 95 | 1 | B2 | >20 |
| PA29B | 12 | 4 | B2 | 45 |
| PA30B | 69 | 17/77 | D | 25 |
| PA31B | 607 | 173 | A | >20 |
| PA32B | 636 | 21 | B2 | 175 |
| PA33B | 973 | 11 | D | >20 |
| PA37B | 73 | 6 | B2 | >20 |
| PA38B | 537 | 75 | B2 | >20 |
| PA41B | 95 | 2 | B2 | 100 |
| PA42B | 420 | 46 | D | >20 |
| PA44B | 12 | 4 | B2 | >20 |
| PA45B | 95 | 2 | B2 | 4000 |
| PA47B | 95 | 1 | B2 | 21 |
| PA48B | 73 | 25 | B2 | 3400 |
| PA50B | 95 | 2 | B2 | 180 |
| PA51B | 131 | 16 | B2 | 65 |
| PA52B | 73 | 25 | B2 | 1000 |
| PA53B | 973 | 21 | D | >20 |
| PA54B | 681 | 9 | B2 | >20 |
| PA55B | 12 | 5 | B2 | 130 |
| PA56B | 2800 | 46 | B2 | >20 |
| PA57B | 372 | 83 | B2 | 3400 |
| PA58B | 295 | N/A | B1 | 30 |
| PA59B | 69 | 11 | D | 90 |
| PA60B | 127 | 6 | B2 | 14000 |
| PA63B | 95 | 1 | B2 | 4860 |
| PA65B | 69 | 17/77 | D | >20 |
| PA66B | 127 | 6 | B2 | >20 |
| PA67B | 131 | 25 | B2 | >20 |
| PA70B | 95 | 1 | B2 | 400 |

* Yellow Highlighted strains had associated sera containing inhibitory antibody, blue highlighted were tested and have no inhibitory antibody
